# Season-dependent phenological responses and implications for co-occurring plants and butterflies under climate anomalies and extreme weather events

**DOI:** 10.1098/rspb.2026.1237

**Published:** 2026-10-07

**Authors:** Hong-Geun An, Moo-Sung Kim, Jung-Dal Sohn, Dong-Hak Kim, Ji-Young Jung, Eunsuk Kim, Il-Kwon Kim

**Affiliations:** ^1^ Department of Environmental and Energy Engineering, Gwangju Institute of Science and Technology, Buk-gu, Gwangju, Republic of Korea; ^2^ Division of Education, Honam National Institute of Biological Resources, Mokpo-si, Jeollanam-do, Republic of Korea; ^3^ Division of Forest Biodiversity, Korea National Arboretum, Pocheon-si, Gyeonggi-do, Republic of Korea; ^4^ Division of Forest Biodiversity Conservation, Korea National Arboretum, Pocheon-si, Gyeonggi-do, Republic of Korea; ^5^ Division of Forest Biological Resources Utilization Center, Korea National Arboretum, Yangpyeong Gyeonggi-do, Republic of Korea

**Keywords:** phenology, climate anomaly, extreme weather event, butterfly, pollinator, gwangneung forest

## Abstract

Despite extensive work on climate-driven phenological shifts, research has focused primarily on early-season events, single metrics and gradual shifts in mean climate, limiting insights into phenological responses across the full annual cycle. Using a 17-year dataset (2009–2025) from an old-growth forest, we examined how co-occurring plants and butterflies responded to climate anomalies and extreme weather events (EWEs) across multiple phenological metrics and seasons, and whether variation in their phenological overlap had ecological implications. Including EWEs substantially improved model performance, particularly for butterfly phenology, and revealed shifts opposite to those driven by climate anomalies, indicating distinct effects on phenology. Responses were also markedly season- and taxon-specific. When plants and butterflies diverged, the pattern varied between seasons: in spring, differences were primarily in magnitude, whereas in summer, they more often involved opposite directions (i.e. one taxon advanced while the other was delayed). However, these differential responses did not translate into changes in phenological overlap in response to climate anomalies or EWEs. Greater overlap was nonetheless associated with higher butterfly diversity, and this association was season-dependent. Our findings suggest that both phenological responses and their ecological consequences may depend strongly on seasonal context, highlighting the importance of considering seasonality and EWEs alongside mean climatic conditions when assessing phenological shifts under climate change.

## 1. Introduction

Phenology, the timing of recurring life cycle events, is driven by environmental cues such as temperature and precipitation; however, climate change alters these cues, thereby shifting the phenologies of many organisms [1,2]. Phenological shifts often differ in direction and magnitude among taxonomic groups, potentially leading to phenological mismatches that disrupt ecological interactions, including mutualistic relationships, food webs and host–parasite interactions, which are fundamental to the functioning of terrestrial ecosystems [3,4]. For example, Kudo & Cooper [5] showed that earlier snowmelt advanced flowering onset more strongly than bumblebee emergence, resulting in phenological mismatch and reduced seed production owing to insufficient pollination services. In turn, pollinators may experience reduced access to floral resources, which could negatively affect their population fitness [6]. Such mismatches may disrupt species interactions and ultimately affect population dynamics and biodiversity [7,8].

To date, research on phenological shifts has largely focused on responses to gradual changes in mean climate conditions (e.g. mean temperature), particularly on early-season events, to evaluate the ecological consequences of climate change [9–11]. However, climate change manifests not only as gradual shifts in mean conditions but also as an increase in the frequency and severity of extreme weather events (EWEs), such as heatwaves, cold snaps, heavy rainfall and droughts [12]. Although studies explicitly incorporating EWEs remain limited, emerging evidence suggests that EWEs can affect phenology to a similar extent as mean climate variables and may remain important drivers even after accounting for mean climatic conditions [10–13]. Furthermore, research has largely focused on spring events, leaving other seasons such as summer and autumn comparatively understudied [9–14]. Nevertheless, different seasonal environmental cues may lead to season-specific phenological responses, suggesting that spring findings may not be generalizable to other seasons [9–15,16].

Butterflies are frequent visitors of flowering plants and represent one of the most extensively monitored insect groups worldwide [17]. They are considered an ideal study system for investigating the impacts of climate change because their life cycles, developmental stages and activity patterns are strongly regulated by climate conditions [18,19]. Although butterflies are known to transport pollen less effectively than other pollinator groups (e.g. bees [20]), they tend to fly longer distances between flowers, which may promote outcrossing and potentially contribute to genetic diversity [21]. Consequently, butterflies rely on the availability of floral nectar resources, while plants may benefit from butterfly-mediated pollen transfer. This association offers a valuable opportunity to examine how climate change affects phenological synchrony between interacting taxa and the resulting ecological consequences. However, many phenology studies of interacting species have relied on single phenological metrics, such as onset or peak timing, rather than the full phenological period or temporal overlap between interacting species, which may not accurately capture phenological mismatches between species [22,23]. This is partly because simultaneously monitoring multiple phenological metrics of interacting species across seasons is labour-intensive and requires long-term observational efforts. Together, these gaps limit our ability to understand how climate change drives phenological shifts, temporal overlap and their ecological consequences across the annual cycle.

To address these gaps, we used a 17-year dataset (2009–2025) from Gwangneung Forest, South Korea, an old-growth forest strictly conserved for over 560 years, to investigate how climate anomalies and EWEs affect multiple phenological metrics of co-occurring plants and butterflies across spring, summer and autumn. We also quantified phenological overlap, the number of days during which plant flowering and butterfly flight periods co-occurred, to examine the ecological implications of changes in overlap. Specifically, we examined: (i) how EWEs and climate anomalies drive phenological shifts across spring, summer and autumn in both plants and butterflies; (ii) whether climatic sensitivities in multiple phenological metrics, including onset, peak, offset and duration, differ between co-occurring plants and butterflies; and (iii) how phenological overlap between the two taxa changes with climate and whether such overlap is associated with butterfly community abundance and diversity.

## 2. Materials and methods

### (a) Study site and data collection

Phenological data were collected at Gwangneung Forest, a cool-temperate old-growth forest located in Gyeonggi Province, the central Korean Peninsula (40–620 m a.s.l., 37°42′–37°45′ N, 126°00′–126°02′ E) [24]. The mean annual air temperature is 10.4°C, ranging from –5.5°C in January to 24.4°C in August, and the mean annual precipitation is 1388 mm, with approximately 65% of precipitation occurring during the summer monsoon season (June–August), peaking in July (418 mm). This area has been strictly preserved as a royal court forest for approximately 560 years since the fifteenth century. Currently, it comprises old-growth (602.3 ha) and experimental (463 ha) forests, and is designated as a UNESCO biosphere reserve under governmental management. The forest harbours the highest biodiversity per unit area in South Korea, with 6564 species recorded across 12 taxonomic groups [25]. Its long-term protection has resulted in minimal anthropogenic disturbance and a relatively stable ecological community, making it well-suited for examining climate-driven phenological responses.

Plant phenology was monitored from 2009 to 2025 for 83 plant species at 74 fixed observation points within a subset of Gwangneung Forest. At each point, one to three individually marked plants per species were repeatedly monitored across years. During each weekly survey (March–November), two to four trained observers visually assessed the flowering stage in the field following standardized criteria to ensure data quality, and photographic records were taken at each survey and subsequently used to verify field assessments. A total of 16 phenological phases were recorded. In this study, we primarily focused on four phenological metrics, defined according to these standardized criteria: (i) onset (first flowering date), the day when petals begin to open and the pistils or stamens become visible; (ii) peak, the date when more than 50% of flowers have fully bloomed; (iii) offset (end of flowering date), the date when more than 90% of flowers have fallen or senesced; and (iv) duration, calculated as the number of days between onset and offset.

Butterfly surveys were conducted from 1998 to 2025 by a single lepidopterist along a 2.5 km transect following the Pollard walk method [26], ensuring observer consistency. The transect, established along trails within Gwangneung Forest, passed near most of the plant observation points (supplementary material, figure S1). Surveys were conducted annually from April to October, with each survey commencing at 09.00 h and lasting approximately 3 h, recording butterfly species and abundance. Survey frequency varied before 2016, when the butterfly surveys were generally conducted at approximately biweekly intervals, and was standardized to a weekly interval thereafter. A total of 130 butterfly species were recorded during the survey period. Although butterfly data were available from 1998, only the 2009–2025 period was used for phenological analyses to align with the plant phenology data.

To assess potential phenological mismatches between plants and butterflies, analyses were restricted to plant species visited by butterflies and nectarivorous butterfly species, based on direct field observations and an extensive literature review. Species with records in 5 or more years were retained, resulting in 29 of the 83 monitored plant species (monitored at 26 fixed observation points; supplementary material, figure S1) and 37 of the 130 recorded butterfly species for analysis (tables S1 and S2). The final phenological dataset comprised 511 plant species–year records and 567 butterfly species–generation–year records.

### (b) Estimation of phenological metrics

As many butterfly species in Gwangneung Forest are multi-voltine, we estimated generation cutoffs for each species to examine phenological metrics across seasons. Survey records were converted to day of year (DOY). Generation cutoffs were then estimated using weighted kernel density estimation. For each species, abundance records were pooled across years (1998–2025), excluding years with fewer than 10 survey days (1999, 2001, 2003, 2004 and 2014; five in total) to ensure reliable delineation of generations. Pooled records were standardized by annual total abundance to reduce interannual differences and weighted by annual survey effort (number of survey days in each year) to account for variation in survey intensity. We then identified the valleys between peaks in the pooled density curve to determine generation boundaries for each species. Based on these boundaries, individual records were assigned to generations within each year. Phenological metrics—onset, peak and offset—were estimated as the 5th, 50th and 95th quantiles [27] for each species–generation combination in each year. Duration was calculated as the number of days between onset and offset. Because survey frequency varied across the study period (generally biweekly before 2016 and weekly thereafter), we additionally tested whether this difference in survey interval could have affected our phenological metric estimates. Specifically, we repeatedly thinned the weekly butterfly records from 2016–2025 to simulate biweekly sampling at different starting points and re-estimated onset, peak and offset using the same method. The results were similar to those using weekly estimation, with median absolute differences of 0 days for onset, peak and offset, and mean absolute differences smaller than the natural interannual variation in these metrics (supplementary material, table S3), suggesting that our phenological estimates are robust to variation in survey intervals.

To compare phenological responses across seasons, plant and butterfly species–generation combinations were assigned to a season based on the median peak DOY across all years. Species were classified as spring (March–May), summer (June–August) or autumn (September–November) based on their median peak DOY.

### (c) Climate variables

Climate data were obtained from a Korea Meteorological Administration weather station located within 1 km of the study site for the period 1997–2025. Daily temperature and precipitation data were used to determine EWEs and climate anomalies.

EWEs were defined following Li *et al.* [10]. We identified four types of EWEs (extreme warm, cold, wet and dry). Extreme warm days were defined as days on which the 7-day centred rolling mean of daily maximum temperature exceeded the long-term mean (1997–2025) for the corresponding calendar day by more than 2 standard deviations (SDs), whereas extreme cold days were defined as days on which the 7-day centred rolling mean of daily minimum temperature was more than 2 SDs below the corresponding long-term mean. Extreme wet and dry days were determined using the modified rainfall anomaly index (mRAI) [10,28]. The mRAI quantifies whether the 7-day accumulated precipitation for a given calendar day is unusually wet or dry relative to the long-term precipitation distribution for that date. Days with mRAI ≥ 2 were classified as extreme wet days, whereas days with mRAI ≤ −2 were classified as extreme dry days. For onset and peak phenology, EWEs were counted as the number of extreme days within the 60 days preceding each phenological date. For offset, EWEs were quantified as the proportion of extreme days between onset and offset. For duration, EWEs were quantified as the proportion of extreme days within the period from 60 days before onset to the offset date (i.e. 60 days + duration). EWEs for offset and duration were logit-transformed before analysis. For each target year, long-term baseline values for each calendar day were calculated by excluding that year from the baseline period (1997–2025).

Climate anomalies were used to enable direct comparisons of phenological responses across seasons, as mean climate conditions differ substantially among seasons. Previous studies have also shown that anomalies can better explain phenological responses than climate means [27]. Temperature anomalies were calculated as deviations from the long-term mean for each phenological metric. Precipitation anomalies were calculated as log-transformed ratios of accumulated precipitation to the long-term mean precipitation owing to the asymmetric distribution of precipitation data. Climate anomalies were calculated using the same baseline approach and time windows as those used for the corresponding EWEs for each phenological metric.

### (d) Statistical analysis

All statistical analyses were conducted using R version 4.5.1 (R Foundation for Statistical Computing, Vienna, Austria) [29]. To investigate how plant flowering and butterfly flight phenology respond to both EWEs and climate anomalies, we employed Bayesian hierarchical models separately for each phenological metric (onset, peak, offset and duration) using the brms package (version 2.23.0 [30]). For each metric, we fitted three models: an anomaly-only model including temperature and precipitation anomalies, an EWE-only model including the four EWEs, and a full model including both anomalies and EWEs. All predictors were standardized to have a mean of 0 and a standard deviation of 1 using z-score scaling. This standardization was performed on the pooled dataset combining both taxa prior to fitting the taxon-specific models, to ensure comparability of effect sizes both across predictors and between taxa. We then assessed multicollinearity among predictors using variance inflation factors (VIFs). All VIF values were <5 in both plant and butterfly models, suggesting that multicollinearity was not a concern (supplementary material, table S4).

Each phenological metric was expressed as DOY and modelled as a function of climate predictors. Separate models were fitted for plants and butterflies. Season was included as an interaction term with all predictors to evaluate potential differences in phenological responses to climate variables among seasons. Random intercepts were included for species and year, and species-specific random slopes were fitted separately for each climate variable. For plant models, random intercepts for individual plants were additionally included to account for repeated observations of the same individuals across years. For butterfly models, the log-transformed number of survey days was included as a covariate to account for variation in sampling effort across years. In the full model, random slopes were included only for climate anomaly variables (temperature and precipitation) rather than for all predictors, as including random slopes for all predictors would increase model complexity and lead to convergence issues. To assess whether plants and butterflies differ in their phenological responses to climate variables across seasons, we extracted season-specific posterior slope distributions from each model and calculated the difference in slopes between plants and butterflies, with 95% Bayesian credible intervals (BCIs). The relative predictive power of anomaly-only, EWEs-only, and full models was compared using leave-one-out cross-validation, with the top-ranked model assigned a Δexpected log predictive density (ELPD) of 0 and lower values indicating poorer predictive performance. Differences were considered substantial when the absolute ΔELPD exceeded twice its standard error. All prior distributions were weakly informative. Normal priors were specified for the intercepts (Normal(165, 150) for onset, peak and offset; Normal(35, 50) for duration) and for slopes (Normal(0, 30)). We ran five parallel Markov chain Monte Carlo (MCMC) chains for 5000 iterations, discarding the initial 2000 iterations as warm-up. To assess convergence, we used the Gelman–Rubin diagnostic (R-hat [31]), with values less than 1.01 indicating convergence.

To estimate the phenological overlap period between co-occurring flowering plants and butterflies, we first modelled onset and offset dates separately for plants and butterflies as a function of year–season interactions, with year treated as a factor. Species was included as a random intercept; plant models additionally included individual plants as a random intercept, whereas butterfly models included log-transformed survey effort as a covariate. Temporal overlap for each year–season combination was then calculated as the number of days between the later onset and the earlier offset of the two taxa. To examine the relationship between climate and phenological overlap, climate anomalies and EWEs were calculated from 60 days before the earlier onset to the later offset of the two taxa. EWEs were quantified as the proportion of extreme days within this window and logit-transformed before analysis. Climate variables for overlap analyses were calculated using the same baseline approach described above. The effect of each climate variable on temporal overlap was estimated using separate Bayesian models for each climate variable, with season included as an interaction term and year as a random intercept. To assess whether variation in phenological overlap was associated with butterfly communities, we calculated butterfly abundance and diversity for each year–season combination. Abundance and diversity were calculated using all 80 nectarivorous butterfly species observed during the study period. Because the limited number of records for 43 of the 80 species did not allow reliable delineation of generation boundaries, seasonal assignment for all 80 species was based on survey month rather than peak activity timing. Abundance was calculated as the total number of individuals per year–season combination, with the log-transformed number of survey days included as an offset. Species diversity was estimated using coverage-based rarefaction (coverage = 0.80) using the iNEXT package [32]. Hill numbers (*q* = 1, corresponding to Shannon diversity; *q* = 2, corresponding to inverse Simpson diversity) were then derived [33]. Separate Bayesian models were constructed for abundance and diversity, with temporal overlap between plants and butterflies in the previous year (*t* − 1) as the predictor, season as an interaction term and year as a random intercept. Abundance was modelled using a negative binomial distribution. To evaluate the robustness of our results, we additionally conducted analyses using only the 37 species included in the phenology analysis, for which seasonal assignment was based on generation delineation derived from peak activity periods identified from multi-year aggregated abundance distributions (supplementary material, figure S2). All priors were weakly informative (Normal(20, 30) for intercepts and Normal(0, 30) for slopes in models estimating the effect of climate variables on temporal overlap; Normal(3, 3) for intercepts and Normal(0, 2) for slopes in abundance models; and Normal(0, 10) for intercepts and Normal(0, 5) for slopes in diversity models), and MCMC settings were identical to those described for the previous models.

## 3. Results

### (a) Model comparison among anomaly-only, EWEs-only and full models

Models incorporating EWEs generally showed the highest or comparable predictive performance across phenological metrics and taxa (table 1). The anomaly-only model showed substantially lower predictive performance for butterfly onset, peak and offset, suggesting that climate anomalies alone may be insufficient to predict butterfly phenology. For plant flowering duration, the EWEs-only model performed best, with both the anomaly-only and full models performing substantially worse. Overall, the predictive importance of climate anomalies and EWEs was phenophase- and taxon-specific.

**Table 1.** Relative predictive performance of anomaly-only, extreme weather events-only and full models across multiple phenological metrics and taxa, based on leave-one-out cross-validation.

phenology	taxa	anomaly only	EWEs only	full model
onset	plant	**0**	−8.4 (± 18.1)	−4.9 (± 15.2)
	butterfly	−68.5 (± 16.0) *	−30.9 (± 11.9) *	**0**
peak	plant	−2.8 (± 17.3)	**0**	−9.8 (± 18.6)
	butterfly	−109.1 (± 16.9)*	**0**	−12.2 (± 14.3)
offset	plant	−16.4 (± 10.7)	−27.4 (± 12.7) *	**0**
	butterfly	−28.4 (± 12.5)*	−3.0 (± 6.7)	**0**
duration	plant	−61.6 (± 24.3) *	**0**	−75.8 (± 23.6) *
	butterfly	−4.4 (± 5.4)	−3.0 (± 4.7)	**0**

Values represent Δexpected log predictive density (ELPD) (± SE) relative to the top-ranked model, which was assigned a value of 0 (bold). Asterisks (*) indicate models with substantially lower predictive performance, defined as |ΔELPD| exceeding twice its standard error. EWEs, extreme weather events.

### (b) Phenological responses to climate anomalies and EWEs across seasons

Plants and butterflies exhibited season-specific phenological responses to both climate anomalies and EWEs (figure 1). In spring, both taxa generally responded in the same direction across climate variables. Warmer-than-average temperatures, positive precipitation anomalies and more extreme dry days tended to advance onset and peak timing (figure 1a,b), whereas more extreme cold days advanced offset (figure 1c). In butterflies, flight duration shortened under positive precipitation anomalies but lengthened with increasing extreme wet days (figure 1d). In summer, however, plants and butterflies tended to respond in opposite directions to climate variables. Positive precipitation anomalies advanced offset timing in plants but delayed it in butterflies (figure 1c). Likewise, in plants, more extreme warm days tended to advance onset and peak timing, and more extreme wet days tended to advance onset timing, whereas the corresponding phenological timing was delayed in butterflies (figure 1a,b). Nevertheless, both taxa also responded in the same direction to several climate variables in summer. Positive temperature anomalies tended to delay onset and peak timing in both taxa, whereas more extreme cold and wet days advanced offset timing and more extreme dry days advanced onset timing (figure 1a,c). Responses to several climate variables were taxon-specific in summer. In plants, positive temperature anomalies advanced offset timing, and more extreme dry days advanced offset timing and shortened flowering duration (figure 1c,d). In butterflies, more extreme cold days delayed onset and peak timing, whereas more extreme dry days advanced onset and peak timing (figure 1a,b). Positive precipitation anomalies also advanced onset timing, while more extreme wet days delayed peak timing. In autumn, an increasing number of extreme cold days advanced peak timing and shortened flight duration in butterflies, and more extreme dry days tended to delay peak timing in both taxa (figure 1b,d).

**Figure 1.**
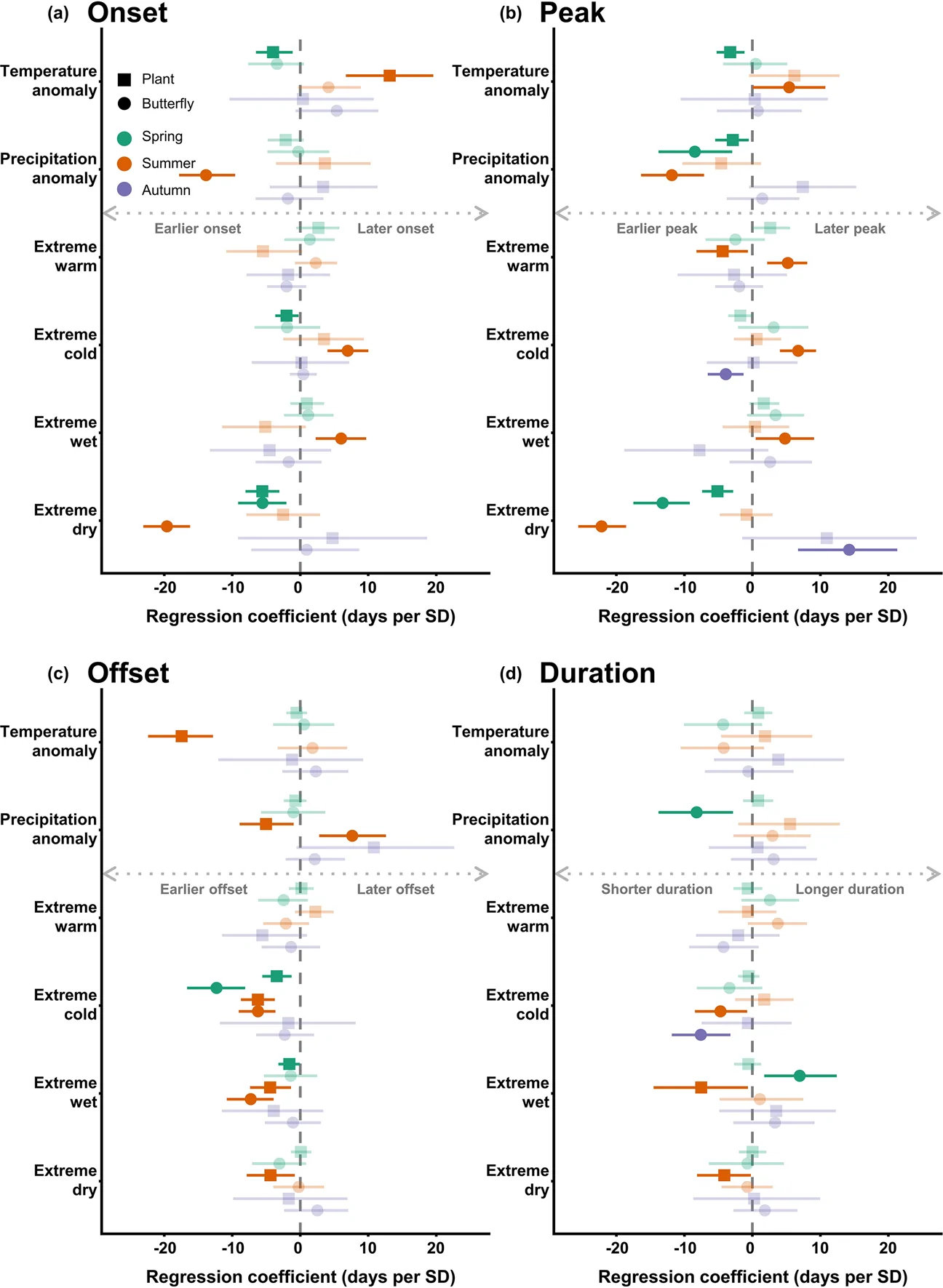
Estimated regression coefficients for the effects of climate anomalies and extreme weather events on plant and butterfly phenology across seasons, including (a) onset, (b) peak, (c) offset and (d) duration. Squares and circles represent plants and butterflies, respectively, and horizontal lines indicate 95% Bayesian credible intervals (BCIs). Colours indicate seasons: spring (green), summer (orange) and autumn (purple). The lighter transparent colours indicate that the 95% BCIs included zero. Estimates are from the full model, including both climate anomalies and extreme weather events as predictors. Dashed arrows indicate the direction of phenological change represented by negative and positive coefficients.

Responses to the same climate variable sometimes reversed direction across seasons for a given phenological metric (figure 1). For example, positive temperature anomalies advanced onset and peak timing in spring but delayed them in summer in both taxa (figure 1b). Similarly, more extreme cold days tended to advance onset in spring but delay it in summer, and more extreme dry days advanced peak timing in spring but tended to delay it in autumn (figure 1a,b). Overall, plants and butterflies generally responded in similar directions to climate anomalies and EWEs, but opposite responses between taxa emerged for some metrics in summer.

### (c) Divergent phenological responses across taxa

Plants and butterflies showed differences in both the direction and magnitude of their phenological responses to climate anomalies and EWEs, which may contribute to phenological mismatches [3,4]. Specifically, these differences were most evident during spring and summer (figure 2). For onset, plants and butterflies showed different phenological shifts for most climate variables in summer, except for extreme cold days (figure 2a). Differences between taxa in peak timing were observed for extreme warm days and extreme dry days in both spring and summer, and for extreme cold days in summer (figure 2b). For offset, taxa responded differently to temperature and precipitation anomalies in summer, and to extreme cold days in spring (figure 2c). For duration, butterfly flight duration was shortened more than plant flowering duration in response to precipitation anomalies in spring and extreme cold days in summer, whereas it was extended more than plant flowering duration in response to extreme wet days in spring (figure 2d).

**Figure 2.**
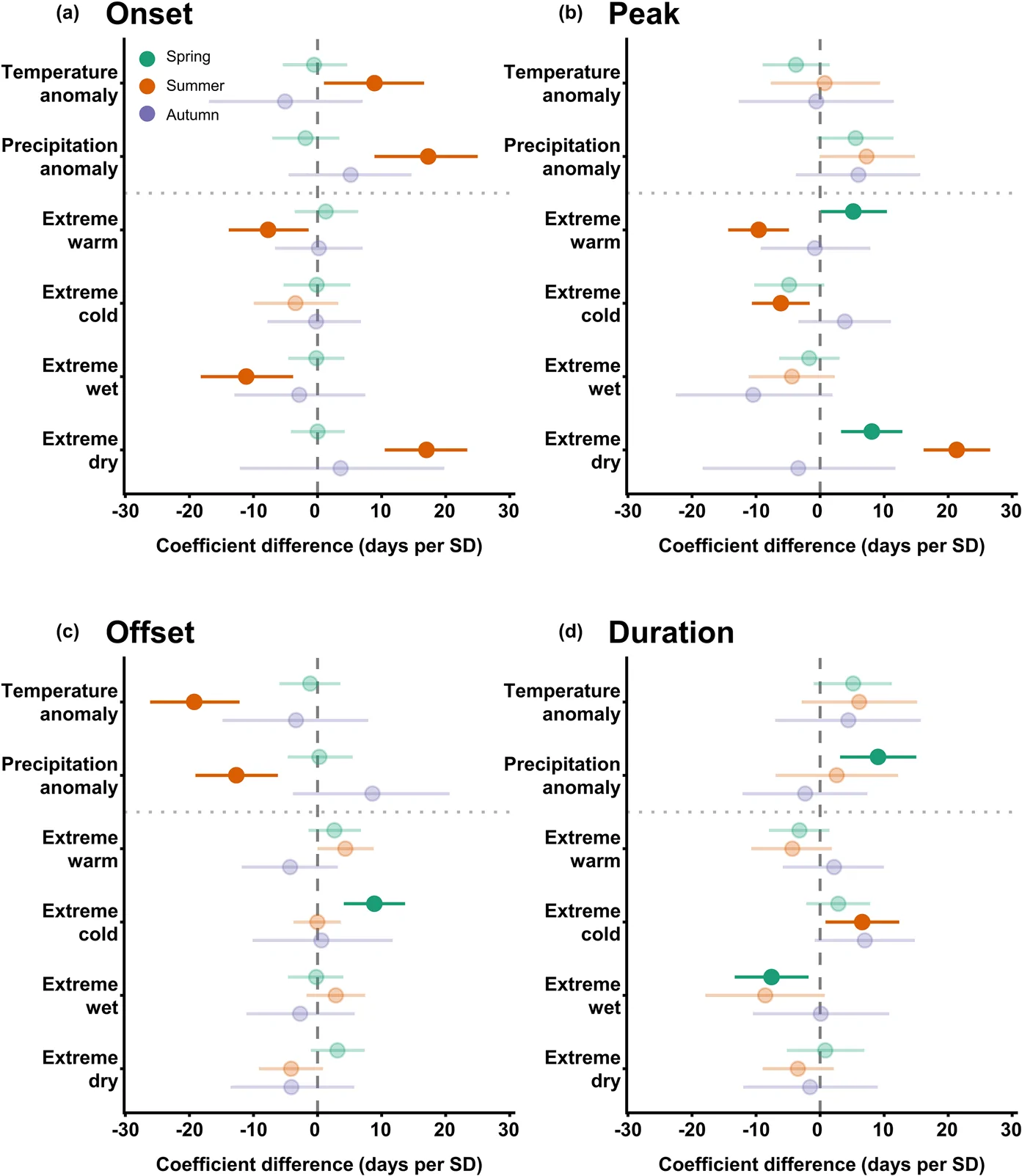
Differences in estimated regression coefficients between plants and butterflies (plant−butterfly) for the effects of climate anomalies and extreme weather events on phenological (a) onset, (b) peak, (c) offset and (d) duration across seasons. Colours indicate seasons: spring (green), summer (orange) and autumn (purple). The lighter transparent colours indicate that the 95% Bayesian credible intervals (BCIs) included zero. Estimated differences are from the full model, including both climate anomalies and extreme weather events as predictors.

Plants and butterflies responded differently to climate anomalies and EWEs in both spring and summer, but the pattern of differences varied across seasons. In spring, both taxa tended to respond in the same direction but differed in the magnitude of their responses. By contrast, differences in summer were more often driven by opposite directional responses between taxa.

### (d) Phenological overlap between co-occurring plants and butterflies

Although plants and butterflies showed differential phenological responses across multiple phenological metrics and seasons, we found no evidence of changes in phenological overlap in response to climate anomalies or EWEs (figure 3). Butterfly abundance was also not associated with phenological overlap across seasons (figure 4). However, greater phenological overlap was positively associated with butterfly diversity, although this association was evident only in spring (95% BCI did not overlap zero; figure 4).

**Figure 3.**
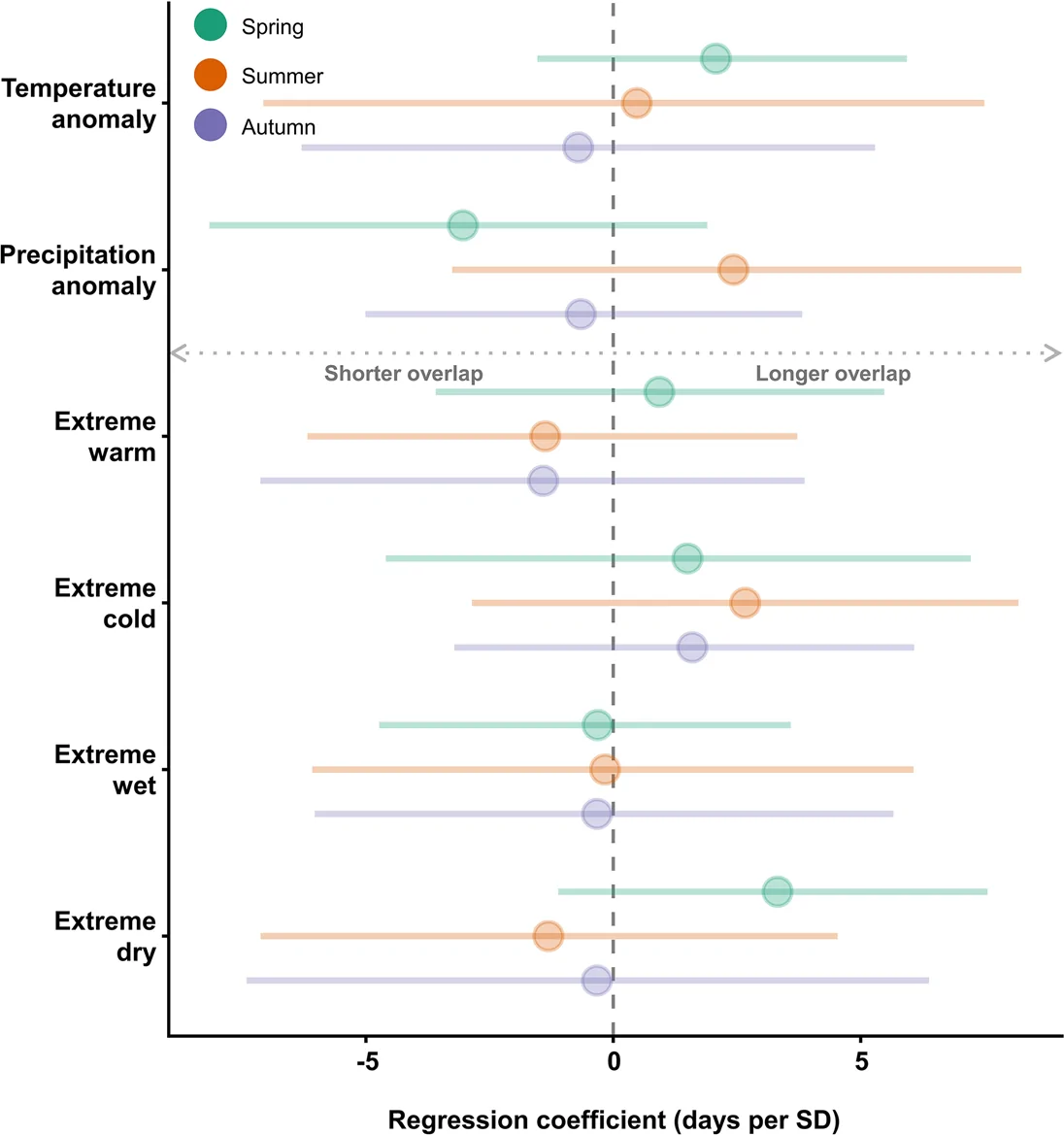
Estimated regression coefficients for the effects of climate anomalies and extreme weather events on phenological overlap between plants and butterflies across seasons. Each climate variable was modelled separately. Circles represent estimated coefficients, and horizontal lines indicate 95% Bayesian credible intervals (BCIs). Colours indicate seasons: spring (green), summer (orange) and autumn (purple). The lighter transparent colours indicate that the 95% BCIs included zero. Dashed arrows indicate the direction of change in phenological overlap represented by negative and positive coefficients.

**Figure 4.**
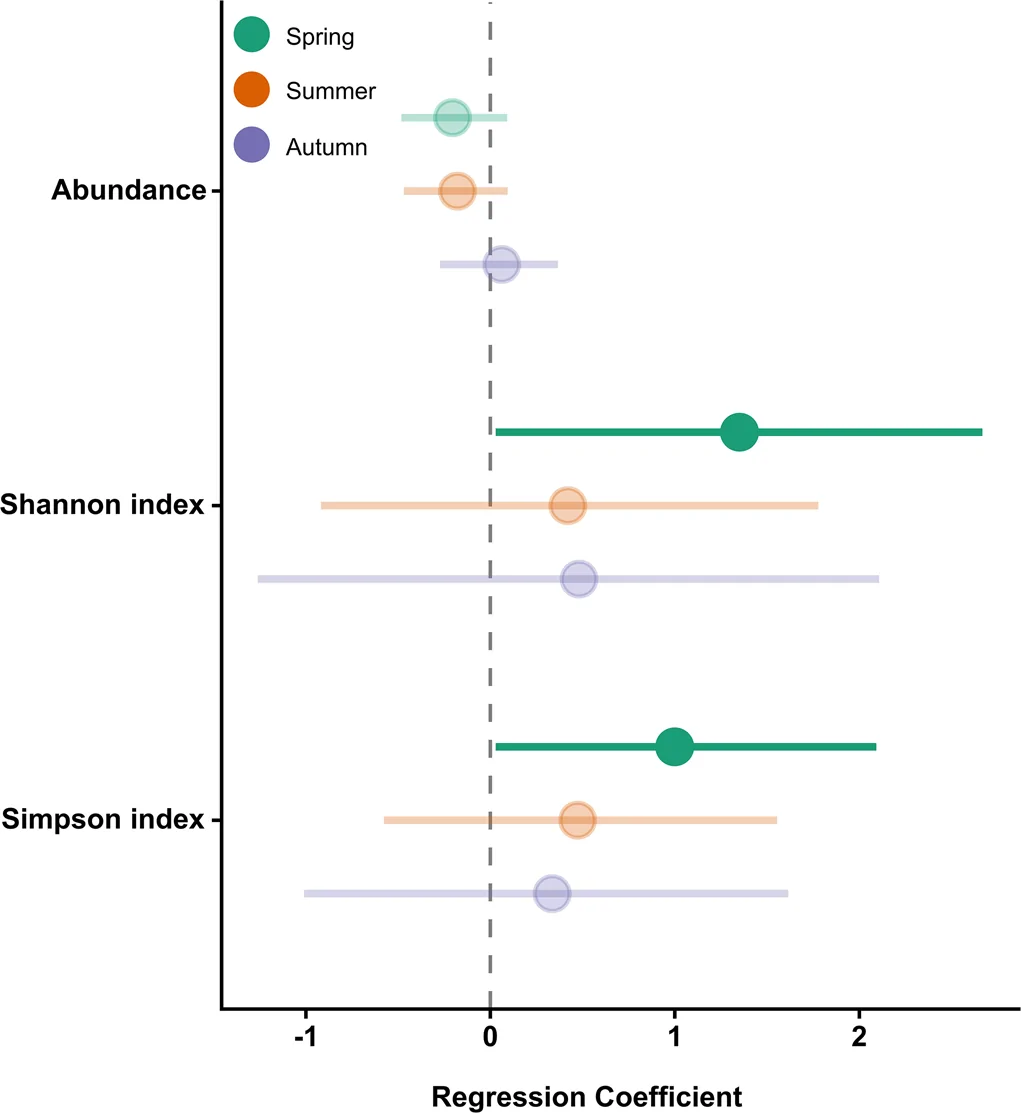
Estimated regression coefficients for the associations between phenological overlap in the previous year (*t* − 1) and butterfly community abundance and diversity across seasons. Circles represent estimated coefficients, and horizontal lines indicate 95% Bayesian credible intervals (BCIs). Colours indicate seasons: spring (green), summer (orange) and autumn (purple). The lighter transparent colours indicate that the 95% BCIs included zero. Abundance and diversity were calculated based on all 80 nectarivorous butterfly species, with seasonal assignment based on survey month.

## 4. Discussion

### (a) Season- and taxon-specific phenological responses to climate anomalies and EWEs across multiple phenological metrics

The ability to explain and predict climate-driven phenological shifts has been largely based on studies of spring phenology and mean climate conditions. Notwithstanding, our results showed that phenological responses were strongly season- and taxon-dependent across multiple phenological metrics, highlighting the need to extend investigations beyond spring for a fuller understanding of climate-driven phenological changes across the annual cycle. Consistent with Li *et al.* [10], EWEs had strong impacts on multiple phenological metrics in both taxa. Notably, the anomaly-only model showed substantially lower predictive performance than models incorporating EWEs, particularly for butterfly phenology. Notably, precipitation anomalies and extreme wet days drove butterfly flight timing in opposite directions in summer across most phenological metrics except for duration: positive precipitation anomalies advanced onset and peak timing but delayed offset, whereas more extreme wet days had the reverse effects on all three metrics. Episodic intense rainfall events may directly impair larval development through physical disturbance and microclimatic cooling, thereby delaying the onset and peak timing of adult flight [34]. By contrast, wetter-than-average conditions may advance flight timing by allowing cumulative precipitation—a key driver of adult emergence—to reach critical thresholds earlier (cf. [35]).

The effects of a given climate variable on phenology were not consistent but varied in direction across seasons. For example, temperatures warmer than the long-term average advanced onset and peak timing in both plants and butterflies in spring but delayed them in summer. These contrasting effects may reflect seasonal differences in the influence of temperature, with warmer conditions accelerating developmental processes in spring but potentially imposing physiological stress during summer. Warmer temperatures during spring may have accelerated development rates and promoted earlier accumulation of the thermal units required for diapause termination in butterflies or flowering in plants, thereby advancing phenological timing in both taxa [36,37]. In summer, however, warming may have acted as a physiological stressor rather than accelerating development, potentially exceeding optimal temperature thresholds for plant reproductive processes [37] and inducing thermal stress in butterflies [38]. These effects may have contributed to delayed phenological timing in both taxa. Unlike in spring and summer, phenological responses in autumn were evident only in butterflies, with extreme cold days advancing peak timing and shortening flight duration, and extreme dry days delaying peak timing. These responses contrasted with those observed in spring and summer: extreme cold days delayed butterfly peak timing in summer, whereas extreme dry days advanced it in both spring and summer. A previous study [10] demonstrated that phenological responses to EWEs can vary with climatic context. Similarly, our results suggest that, because climatic conditions differ substantially among seasons, phenological responses to a given climate variable may also vary depending on seasonal climatic context. In South Korea, seasonal climatic contexts differ markedly. Spring is characterized by rapidly warming but highly variable temperatures and relatively dry conditions; summer by hot, humid weather associated with the East Asian monsoon and typhoons; and autumn by rapid cooling and comparatively dry conditions [39].

One of the key findings of our study was that differences in phenological responses between plants and butterflies varied fundamentally between spring and summer. In spring, differential phenological shifts between the two taxa were driven primarily by differences in magnitude rather than direction. Specifically, both taxa advanced their peak and offset timing in response to extreme dry and cold days during spring. However, butterflies responded more strongly to these extreme events, as the 95% BCI of the estimated slope difference between plants and butterflies did not overlap zero. The advancement of onset and peak timing in response to extreme dry days contrasts with Li *et al.* [10], who reported delayed onset in both plants and Lepidoptera under extreme dry conditions. This discrepancy may partly reflect the seasonal context of drought responses. For example, Castillioni *et al.* [40] found that early-season plant species advanced flowering under reduced precipitation, possibly as an avoidance strategy to complete reproduction before severe drought. By contrast, later-season species delayed flowering under drought, likely reflecting a strategy of suspending reproductive development under stress until conditions improve sufficiently to allow successful offspring establishment [10,40]. A similar advancement in butterfly flight timing under drought conditions has also been reported, possibly because drought is often accompanied by elevated temperatures, which together may have accelerated larval development and advanced adult flight timing [41]. For offset, extreme cold days in spring may include late-frost events that directly damage floral tissues [42,43], potentially advancing flowering offset in plants. In butterflies, cold events may suppress adult flight activity [44,45], making the observed flight offset appear earlier.

Differences between the two taxa were more pronounced in summer, driven largely by opposing directional responses rather than differences in the magnitude of their shifts. These opposing responses were observed in both peak and offset timing, where more extreme warm days advanced flowering peaks but delayed flight peaks, and positive precipitation anomalies advanced flowering offset but delayed flight offset. Paradoxically, plant responses to extreme warm days contrasted with those to positive temperature anomalies in summer, with extreme warm days advancing rather than delaying flowering peak timing, whereas butterfly responses remained consistent, with both delaying flight peak timing. One possible explanation for this divergent pattern is that plants may respond differently to short-term extreme heat events versus prolonged warming [46,47]. For instance, Allen *et al*. [46] showed that continuous warming can produce different developmental outcomes from short-term heat exposure, whereas Wang *et al.* [47] demonstrated that prolonged warming and heat shock induce distinct physiological and metabolic responses in plants. Such differential responses to heat exposure suggest that short-term extreme heat events and prolonged warming may trigger different plant responses, potentially contributing to opposing directional responses to extreme warm days and temperature anomalies. Unlike sessile plants, which cannot escape local thermal extremes, butterflies can behaviourally thermoregulate by relocating to cooler microhabitats [48,49], potentially buffering the effects of extreme heat and contributing to their more consistent responses to extreme warm days and temperature anomalies. For precipitation anomalies, the contrasting directional responses may be attributable to the different proximate effects of rainfall on plants and butterflies. For plants, high precipitation may physically damage reproductive structures [50,51], potentially advancing flowering offset. By contrast, heavy rainfall may temporarily constrain adult butterfly flight activity [50], which may resume once rainfall ceases and thereby delay flight offset.

Our results clearly showed that phenological responses to climate anomalies and EWEs differ substantially across multiple phenological metrics, seasons and taxa. Moreover, EWEs improved predictive performance, particularly for butterfly phenology, and in some cases, drove phenological shifts in opposite directions to those associated with climate anomalies. Given that spring, summer and autumn are characterized by distinct climatic conditions and ecological processes [9], and that climate change is projected to increase the frequency and intensity of extreme events across multiple seasons [12], our findings underscore that investigations of climate-driven phenological shifts should span multiple seasons and incorporate EWEs alongside mean climatic conditions for a fuller characterization of phenological responses under climate change.

### (b) Ecological implications of variation in phenological overlap between plants and butterflies

Unexpectedly, despite the differential phenological responses of plants and butterflies to climate anomalies and EWEs across multiple phenological metrics and seasons, we found no evidence that phenological overlap between the two taxa changed in response to such climate variables. This result is consistent with that of Iler *et al.* [52], who found that flowering and syrphid emergence responded differently to climatic cues, yet temporal overlap between the two groups was maintained. Moreover, butterfly community abundance showed no association with phenological overlap across seasons. This may be partly because our analyses estimated phenological overlap at the community level, where complementary and diverse phenological responses among species may have buffered changes in overall plant–butterfly synchrony [53]. For instance, Kudo *et al.* [54] reported that the effects of phenological mismatch on bumble bee abundance were highly species-specific, with some species decreasing and others increasing, suggesting that diverse responses among species may cancel out at the community level. In other words, community-level responses may reflect the cumulative effects of multiple species-level interactions [55] that differ in both direction and magnitude. Consequently, individual species-level responses may not necessarily align with overall community trends, as also shown by Iler *et al.* [52].

However, greater phenological overlap between plants and butterflies was positively associated with higher butterfly diversity in spring, but not in summer or autumn. This season-specific response may suggest that spring communities are more strongly influenced by phenological asynchrony than those in other seasons. Although the divergent responses of individual species may cancel one another out at the community level, specialist or early-season species that cannot be easily compensated by alternative partners may still be vulnerable to asynchrony [56–58]. In spring, early-season insects may be especially vulnerable to asynchrony owing to limited alternative food resources [56,57], while spring-flowering plants may critically depend on these early-season pollinators for reproduction [5]. Consequently, spring communities may be more sensitive to changes in phenological overlap than those in other seasons. Our findings suggest that the ecological consequences of phenological asynchrony vary across seasons and depend on the ecological context in which interactions occur, such as the availability of alternative partners and the composition of interacting species communities. These results highlight the importance of considering ecological context when evaluating the outcomes of phenological asynchrony.

### (c) Caveats

A key caveat in interpreting our results is that butterflies may not fully represent the responses of other pollinator groups. Climate sensitivity varies substantially across functional groups and even among species within the same group, with phenological responses potentially differing in both magnitude and direction [1]. In particular, many butterfly species are multivoltine and produce several discrete generations during the warm season, whereas other pollinators, especially social hymenopterans, often exhibit continuous and cumulative increases in abundance. These differences in life cycle and population structure among pollinators may therefore lead to different phenological responses to climate anomalies and EWEs, as well as different ecological consequences. For example, similar to other pollinators [59,60], butterflies may advance emergence timing and modify activity patterns in response to warming [19]. However, some species can also increase the number of generations produced annually [61], introducing an additional voltinism-related response that may contribute to the divergent responses to climate change observed among pollinator groups.

## 5. Conclusions

Here, we showed that co-occurring plants and butterflies exhibited markedly different phenological responses to climate anomalies and EWEs across multiple phenological metrics and seasons, with the pattern of divergence varying fundamentally between spring and summer. EWEs improved predictive performance compared with climate anomalies alone, and in some cases drove phenological shifts in opposite directions to those associated with climate anomalies, suggesting that mean climatic conditions alone may be insufficient to understand phenological responses. Despite these differential responses, phenological overlap did not change in response to climate anomalies or EWEs. However, greater phenological overlap was associated with higher butterfly diversity, and this association was season-dependent. Our findings highlight that both phenological responses and their ecological implications may be fundamentally shaped by seasonal context, and underscore the need for further investigation across multiple seasons to improve our ability to predict phenological changes and their ecological consequences under climate change.

## Supplementary Material



## Data Availability

Data and code supporting this article are available from the Open Science Framework [62]. Supplementary material is available online [63].
