# Supplementary material for: Season-dependent phenological responses and implications for co-occurring plants and butterflies under climate anomalies and extreme weather events
Source: Proc Biol Sci. 2026 Oct 7;293(2080):20261237. doi: 10.1098/rspb.2026.1237 (PMC13641889; doi:10.1098/rspb.2026.1237)
Supplement: Supplementary file 1 [file rspb.2026.1237.zip › rspb.2026.1237/rspb20261237_si_001.pdf]

**Electronic Supplementary Material**

For the article published in *Proceedings of the Royal Society B*

(DOI: 10.1098/rspb.2026.1237)

# Season-dependent phenological responses and implications for co-occurring plants and butterflies under climate anomalies and extreme weather events

**Authors:** Hong-Geun An, Moo-Sung Kim, Jung-Dal Sohn, Dong-Hak Kim, Ji-Young Jung, Eunsuk Kim, and Il-Kwon Kim

**Table S1.** List of 29 plant species included in the plant phenology analyses. Season was classified based on the median peak day of year across all study years: spring (March–May), summer (June–August), and autumn (September–November).

| Species | Season |
|---|---|
| *Abeliophyllum distichum* | Spring |
| *Allium microdictyon* | Spring |
| *Berberis koreana* | Spring |
| *Cornus officinalis* | Spring |
| *Corylopsis coreana* | Spring |
| *Erythronium japonicum* | Spring |
| *Forsythia koreana* | Spring |
| *Lindera obtusiloba* | Spring |
| *Primula sieboldii* | Spring |
| *Prunus × yedoensis* | Spring |
| *Rhododendron mucronulatum* | Spring |
| *Rhododendron schlippenbachii* | Spring |
| *Rhododendron yedoense* | Spring |
| *Robinia pseudoacacia L.* | Spring |
| *Scopolia parviflora* | Spring |
| *Sorbus commixta* | Spring |
| *Stephanandra incisa* | Spring |
| *Symplocos sawafutagi* | Spring |
| *Syringa oblata* | Spring |
| *Weigela subsessilis* | Spring |
| *Albizia julibrissin* | Summer |
| *Aruncus dioicus* | Summer |
| *Hemerocallis fulva* | Summer |
| *Koelreuteria paniculata* | Summer |
| *Rhododendron brachycarpum* | Summer |
| *Sanguisorba hakusanensis* | Summer |
| *Stewartia koreana* | Summer |
| *Chrysanthemum boreale* | Autumn |
| *Hylotelephium ussuriense* | Autumn |

**Table S2.** List of 37 butterfly species included in the butterfly phenology analyses. Season was classified based on the median peak day of year across all study years: spring (March–May), summer (June–August), and autumn (September–November). As many butterflies are multivoltine, some species were represented in multiple seasons.

| **Species** | **Season** |
|---|---|
| *Ahlbergia ferrea* | Spring |
| *Aldania thisbe* | Spring |
| *Anthocharis scolymus* | Spring |
| *Araschnia burejana* | Spring |
| *Atrophaneura alcinous* | Spring |
| *Daimio tethys* | Spring |
| *Erynnis montana* | Spring |
| *Luehdorfia puziloi* | Spring |
| *Neptis philyra* | Spring |
| *Neptis philyroides* | Spring |
| *Neptis sappho* | Spring |
| *Papilio bianor* | Spring |
| *Papilio macilentus* | Spring |
| *Papilio xuthus* | Spring |
| *Parnassius stubbendorfii* | Spring |
| *Pieris melete* | Spring |
| *Pseudozizeeria maha* | Spring |
| *Rapala caerulea* | Spring |
| *Tongeia fischeri* | Spring |
| *Argynnis paphia* | Summer |
| *Argynnis vorax* | Summer |
| *Argyronome laodice* | Summer |
| *Argyronome ruslana* | Summer |
| *Celastrina argiolus* | Summer |
| *Cupido argiades* | Summer |
| *Limenitis camilla* | Summer |
| *Limenitis helmanni* | Summer |
| *Neptis alwina* | Summer |
| *Neptis pryeri* | Summer |
| *Neptis speyeri* | Summer |
| *Papilio bianor* | Summer |
| *Papilio maackii* | Summer |

| | |
|---|---|
| *Papilio macilentus* | Summer |
| *Pieris canidia* | Summer |
| *Pieris melete* | Summer |
| *Pieris rapae* | Summer |
| *Tongeia fischeri* | Summer |
| *Argynnis paphia* | Autumn |
| *Argynnis vorax* | Autumn |
| *Argyronome laodice* | Autumn |
| *Argyronome ruslana* | Autumn |
| *Colias erate* | Autumn |
| *Limenitis doerriesi* | Autumn |
| *Papilio xuthus* | Autumn |
| *Parnara guttatus* | Autumn |
| *Pieris melete* | Autumn |
| *Pieris rapae* | Autumn |
| *Pseudozizeeria maha* | Autumn |
| *Vanessa cardui* | Autumn |

**Table S3.** Sensitivity of butterfly phenological estimates (onset, peak, and offset) to survey interval. Weekly butterfly records from 2016–2025 were repeatedly thinned (1000 repeats) to simulate biweekly sampling with different starting points, and re-estimated for the 37 butterfly species included in the phenology analyses. Median and mean absolute differences indicate differences between estimates from the weekly data and the biweekly-thinned data. Mean interannual SD was calculated from weekly data and represents natural interannual variation in each phenological metric.

| Metric | Median absolute difference | Mean absolute difference | Mean interannual SD |
|---|---|---|---|
| Onset | 0 days | 5.8 days | 11.7 days |
| Peak | 0 days | 6.8 days | 12.8 days |
| Offset | 0 days | 6.2 days | 12.4 days |

**Table S4.** Variance inflation factors (VIFs) for climate predictors included in plant and butterfly phenological models. All VIF values were below 5, indicating no multicollinearity among predictors.

| Phenological metric | Predictor | Plant | Butterfly |
|---|---|---|---|
| Onset | Temperature anomaly | 1.86 | 1.91 |
| | Precipitation anomaly | 2.40 | 2.43 |
| | Extreme warm days | 1.67 | 1.51 |
| | Extreme cold days | 1.12 | 1.15 |
| | Extreme wet days | 2.24 | 2.06 |
| | Extreme dry days | 1.58 | 1.90 |
| Peak | Temperature anomaly | 1.87 | 1.96 |
| | Precipitation anomaly | 2.25 | 2.36 |
| | Extreme warm days | 1.66 | 1.52 |
| | Extreme cold days | 1.07 | 1.18 |
| | Extreme wet days | 2.19 | 2.01 |
| | Extreme dry days | 1.47 | 1.58 |
| Offset | Temperature anomaly | 1.54 | 1.48 |
| | Precipitation anomaly | 2.04 | 1.89 |
| | Extreme warm days | 1.43 | 1.37 |
| | Extreme cold days | 1.16 | 1.18 |
| | Extreme wet days | 1.40 | 1.45 |
| | Extreme dry days | 1.74 | 1.39 |
| Duration | Temperature anomaly | 2.08 | 1.94 |
| | Precipitation anomaly | 2.22 | 2.36 |

| | | |
|---|---|---|
| Extreme warm days | 1.59 | 1.53 |
| Extreme cold days | 1.15 | 1.28 |
| Extreme wet days | 2.51 | 2.22 |
| Extreme dry days | 1.37 | 1.48 |

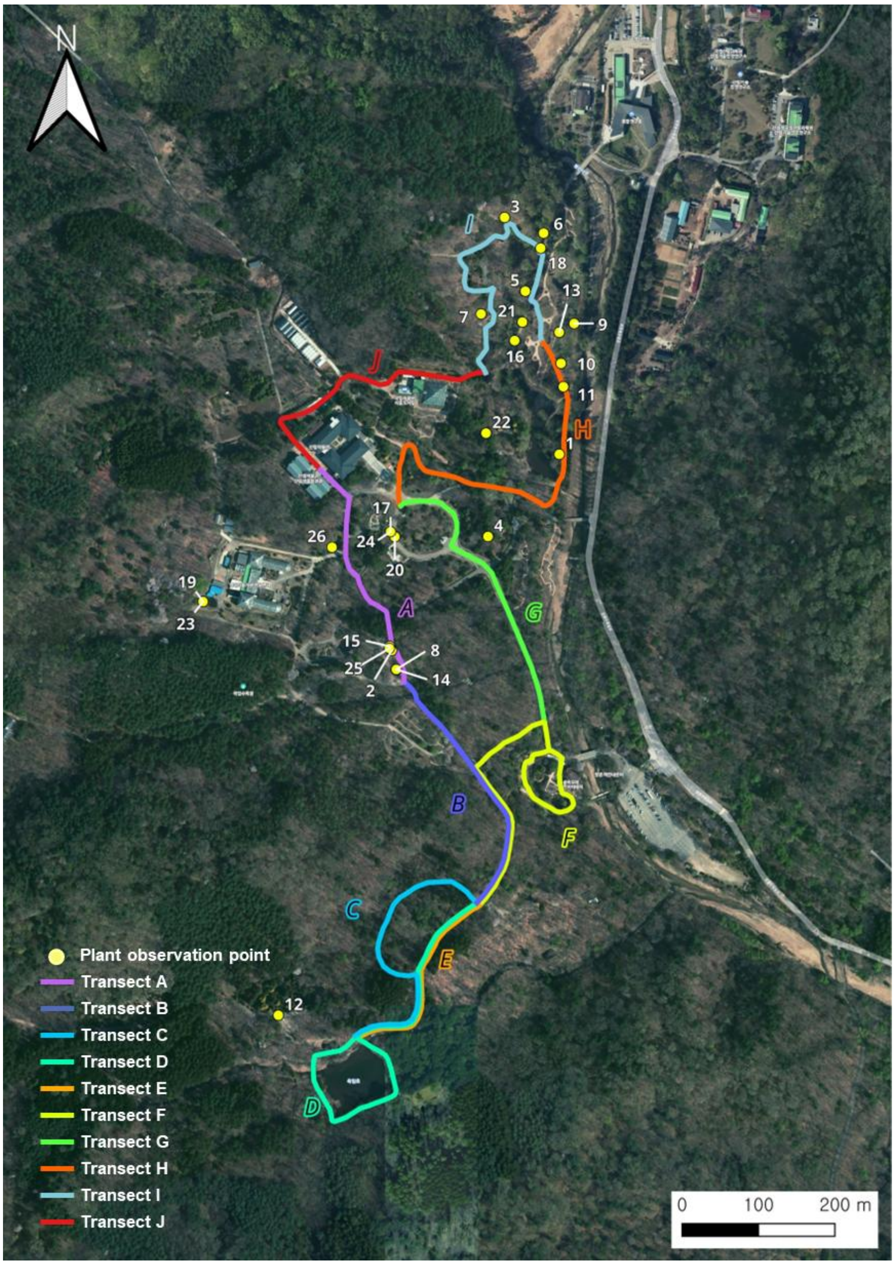


**Figure S1.** Spatial arrangement of the plant observation points and butterfly survey transect within Gwangneung Forest. Yellow circles indicate the 26 fixed plant observation points at which the 29 plant species included in the plant–butterfly phenological analysis were monitored. Coloured lines indicate the 2.5-km butterfly survey transect, divided into 10 sections, labelled A–J. Canopy openness varied among sections: C, E, and J were relatively shaded by dense woody canopies, B was partially shaded, and the remaining sections (A, D, F, G, H, and I) were relatively open.

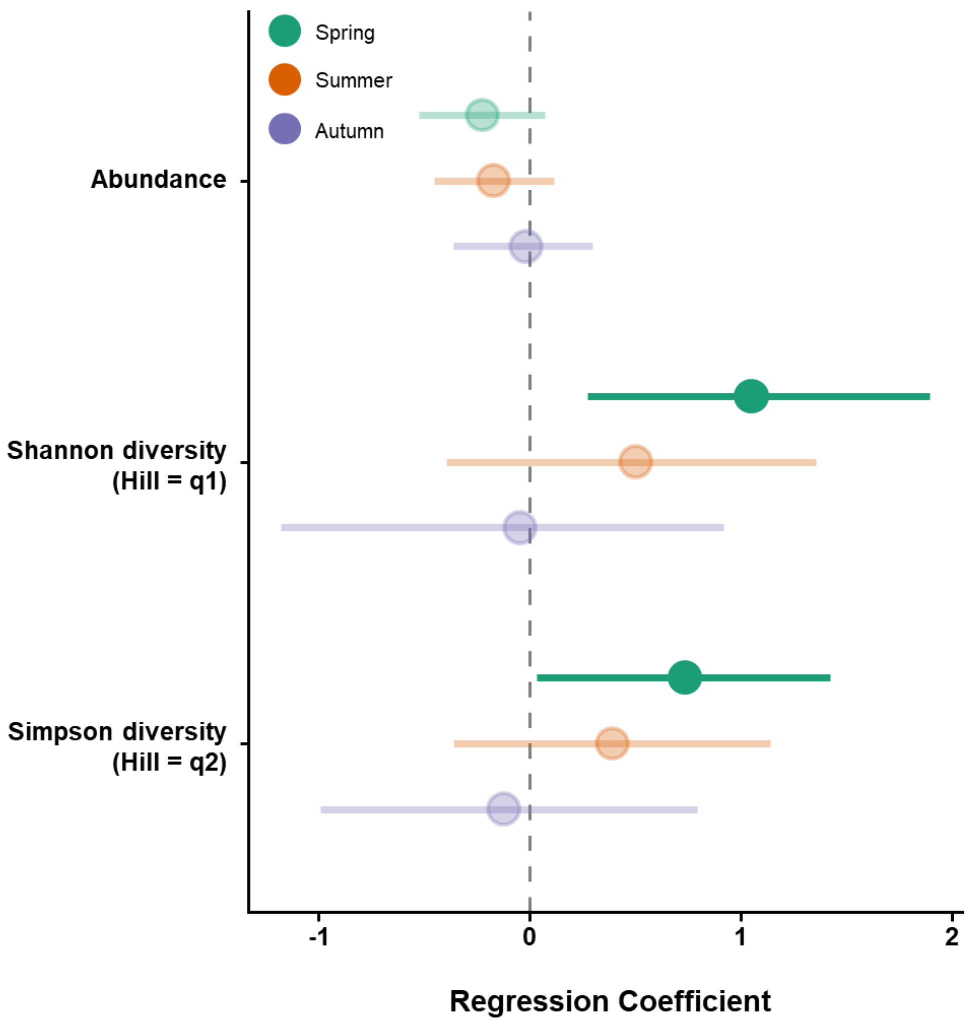


**Figure S2.** Estimated regression coefficients for the associations between phenological overlap in the previous year (t−1) and butterfly community abundance and diversity across seasons. Circles represent estimated coefficients, and horizontal lines indicate 95% Bayesian credible intervals (BCIs). Colours indicate seasons: spring (green), summer (orange), and autumn (purple). The lighter transparent colours indicate that the 95% BCIs included zero. Abundance and diversity were calculated based on the 37 butterfly species included in the phenology analyses. Results were consistent with those obtained using all 80 nectarivorous butterfly species (Figure 4).
